# Jejunoileal GIST: A Rare Case of Transient Intussusception and Gastrointestinal Bleeding

**DOI:** 10.1155/2019/1492965

**Published:** 2019-04-04

**Authors:** Sara Catarino Santos, Cláudia Leite, Helena Pinho, Carlos Casimiro

**Affiliations:** General Surgery Department, Centro Hospitalar Tondela-Viseu, Avenida Rei D. Duarte, 3504-509 Viseu, Portugal

## Abstract

Gastrointestinal stromal tumors (GIST) comprised 0,2% of all GI tumors. They are typically asymptomatic, but can manifest with nonspecific GI symptoms, GI bleeding, or intussusception. The authors report a case of a 55-year-old female patient with hematochezia and a palpable mass on the left lower quadrant. Ultrasound revealed possible intussusception. However, CT scan did not show any signs of lesions or intussusception. On reevaluation, the mass was no longer palpable. The patient had recurrent episodes of hematochezia with need of transfusional support. CT enterography revealed a 20-24 mm jejunoileal lesion. A laparotomy was undertaken with small bowel resection containing the lesion. Histological examination confirmed GIST. GIST presentation as transient intussusception and intermittent GI bleeding is rare. This case report emphasizes the rarity of jejunoileal GIST, its clinical details, diagnostic study, and treatment.

## 1. Introduction

Gastrointestinal stromal tumors (GIST) arise from mesenchymal stromal cells and comprise 0,2% of all gastrointestinal (GI) tract malignancies [1]. These kinds of tumors were first described by Mazur and Clark in 1983 [1, 2]. GIST can originate anywhere along the GI tract. The stomach (40–60%) and small intestine (20%) are the most common locations [2]. Hence, small bowel GIST comprise only 0,04% of all GI tumors [1]. They are typically asymptomatic, but can manifest with nonspecific GI symptoms, GI bleeding, or intussusception [1]. GIST presentation as transient intussusception and intermittent GI bleeding is rare.

## 2. Case Presentation

**Figure 1:**
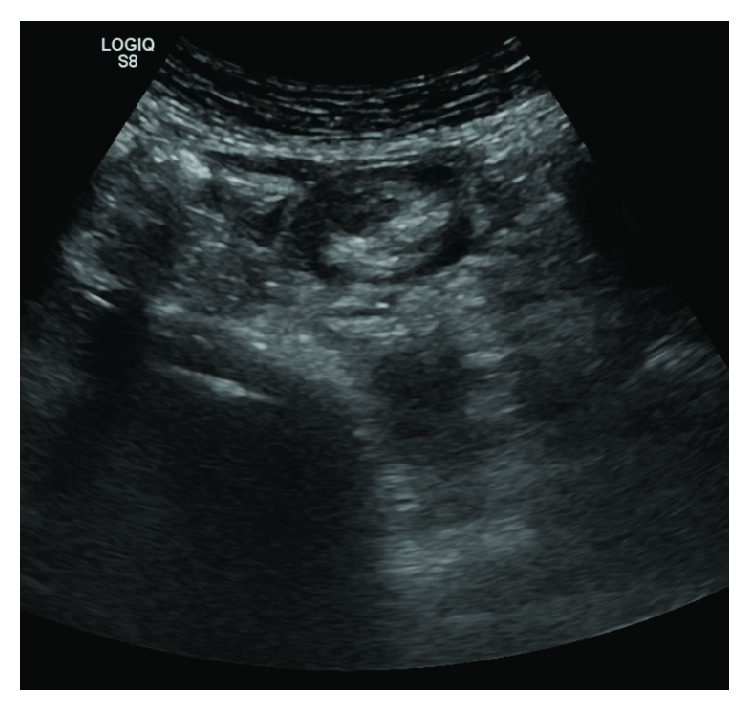
Abdominal ultrasound (possible intussusception).

A 55-year-old Caucasian female patient was admitted in the emergency department with hematochezia and lower abdominal pain. She had no significant prior medical history. On examination, the patient had normal vital signs and a palpable painful mass on the left lower quadrant of the abdomen. Laboratory data revealed a haemoglobin level of 11,8 g/dL. Abdominal ultrasound showed a mass on the left lower quadrant with possible intussusception (Figure 1).

**Figure 2:**
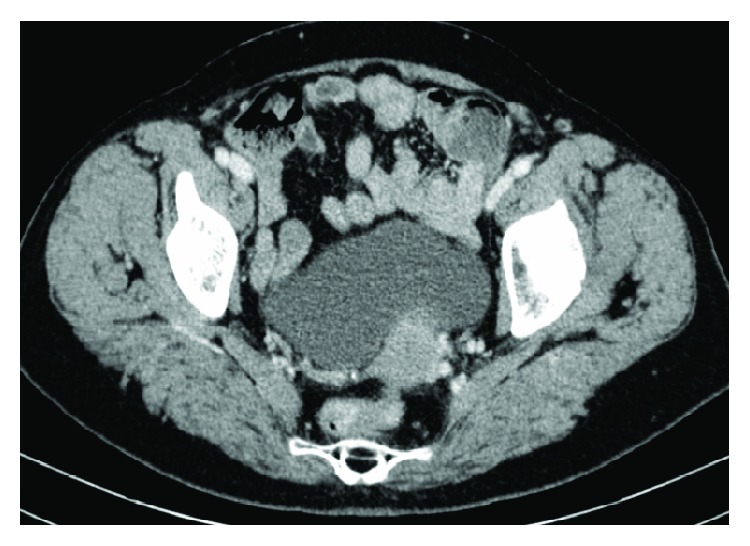
CT scan (no signs of lesions or intussusception).

An abdominal computed tomography (CT) scan was then performed, with rectal and IV contrast, which had no signs of lesions or intussusception (Figure 2). On reevaluation, the mass was no longer palpable, although pain was still present. Subsequently, the patient was admitted on the surgical ward for further investigation.

**Figure 3:**
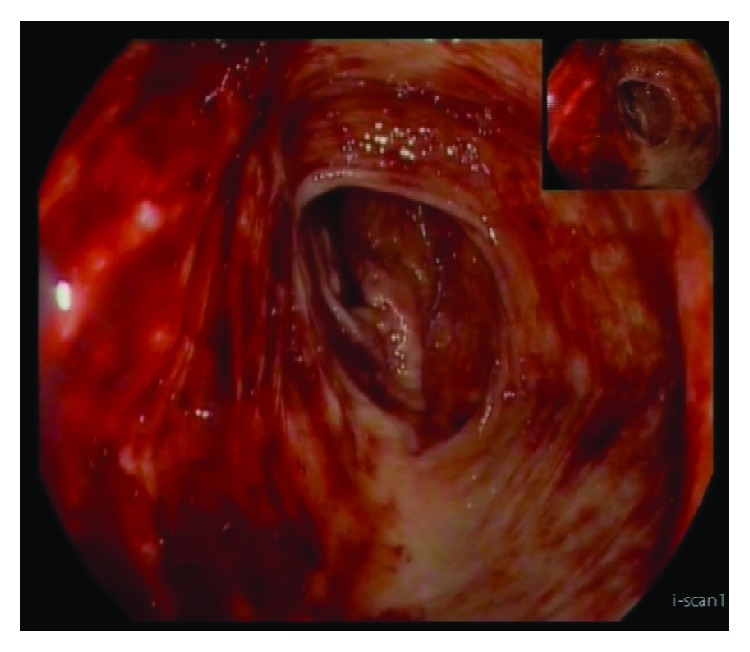
Lower endoscopy (hematic residues with no lesions detectable).

Upper endoscopy was performed which was normal, and lower endoscopy revealed hematic residues but no lesions detectable (Figure 3). Throughout hospital stay, the patient presented intermittent episodes of palpable abdominal mass and intermittent blood loss, with asthenia and syncope.

**Figure 4:**
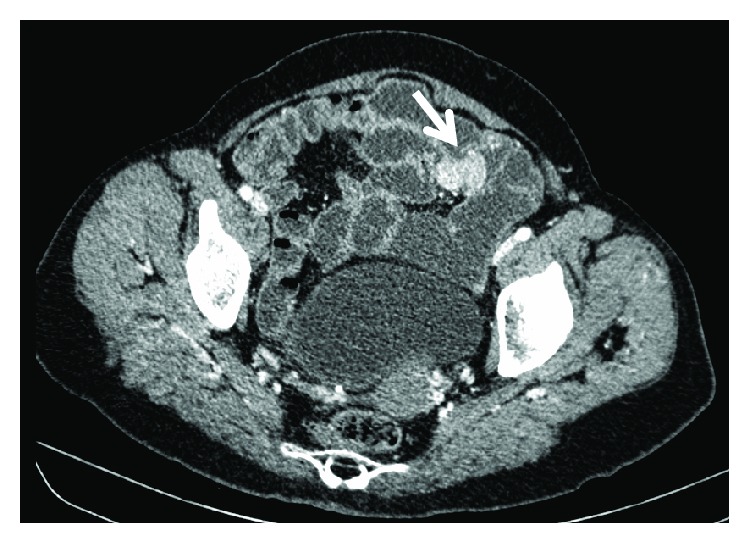
CT enterography—axial CT image with 20-24 mm jejunoileal lesion compatible with GIST.

**Figure 5:**
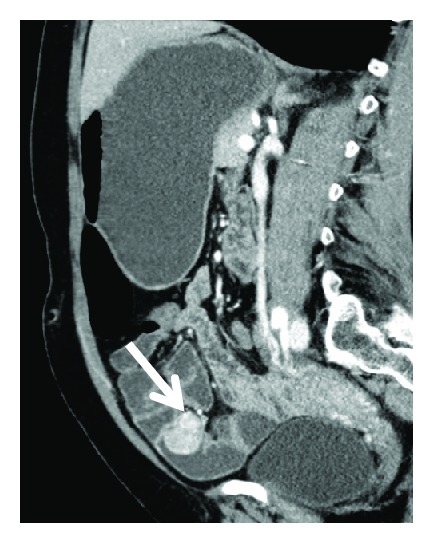
CT enterography—sagittal CT image with lesion compatible with GIST.

**Figure 6:**
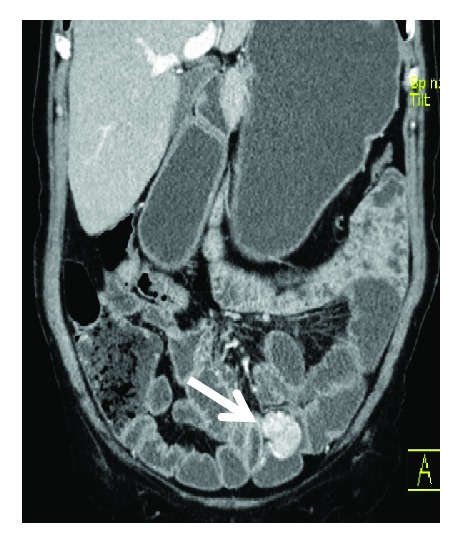
CT enterography—coronal CT image lesion compatible with GIST.

Haemoglobin level dropped to 7,1 g/dL, with need of transfusional support. Repeated lower endoscopy did not show the haemorrhage source. CT enterography was ordered, which revealed a 20-24 mm jejunoileal lesion, compatible with GIST (Figures 4–6).

**Figure 7:**
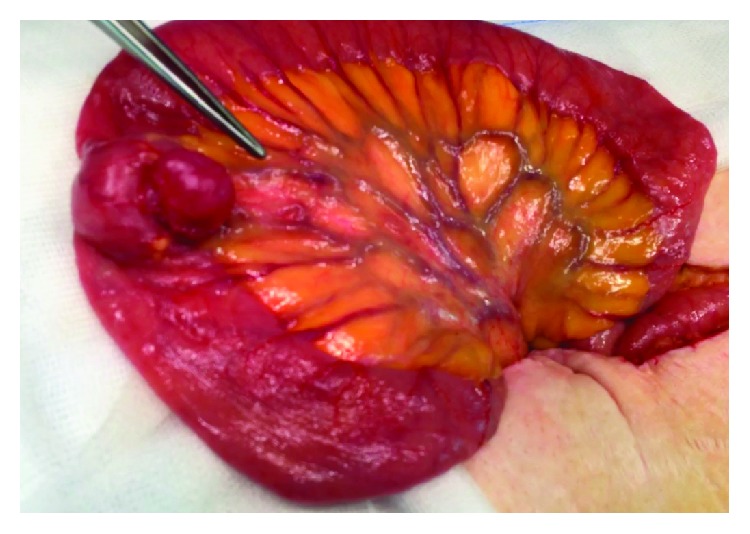
Surgery—jejunoileal lesion compatible with GIST with the endophytic and exophytic component.

Hence, based on known findings, the diagnosis of intermittent GI bleed and transient intussusception due to small bowel GIST was established. A laparotomy was undertaken with small bowel resection containing the lesion (Figure 7). The patient recovered well and was discharged home on the 5th postoperative day. In follow-up consultation, the patient was asymptomatic, without new episodes of GI bleeding.

Histological examination confirmed jejunoileal GIST with 2,6 cm, without necrosis or vascular invasion, with a mitotic index of <5 per 50 high power field (HPF). Thus, it was a low risk GIST, according to the modified National Institute of Health (NIH) method. In multidisciplinary reunion, it was decided to keep the patient only on clinical surveillance.

## 3. Discussion

GIST are rare tumors that occur predominantly in males between 50 and 70 years old [3]. Jejunoileal GIST are typically asymptomatic and may be diagnosed incidentally in imaging studies [1]. However, they may present with nonspecific GI symptoms, GI bleeding, or intussusception [1].

Bleeding from the small bowel accounts for 2-10% of all GI bleedings, and the main causes are vascular abnormalities (70-80%) and tumors (5-10%) [3, 4]. About 28% of GIST present with GI bleeding [1]. Therefore, GIST is rarely the source of small intestinal bleeding and is usually associated with relatively slow bleeding [4]. GI bleeding may be occult, defined as bleeding not visible, or obscure, defined as persistent or recurrent bleeding from which no definitive source has been identified by upper and lower endoscopy [3]. Obscure GI bleeding may be occult (if not visible) or overt (if it manifests with visible blood) [3].

On the other hand, intussusception is a rare phenomenon in adults, which represents 5% of all cases of intussusception [5]. It is defined as the telescoping of a proximal segment of the GI tract, called intussusceptum, into the lumen of the adjacent distal segment of the GI tract, called intussuscipiens [5]. In adults, 90% of intussusceptions are secondary to a pathologic condition, which is malignant in more than 50% of cases [5, 6]. The presenting symptoms are nonspecific and may be intermittent [5], such as abdominal pain, nausea, vomiting, and a palpable abdominal mass [6]. Diagnosis of this condition is challenging. CT scan is considered the most sensitive method to confirm intussusception, but ultrasonography is also useful with the classical feature of the “target” sign. Due to the risk of malignancy in the adult population, surgical resection is usually the treatment of choice [5].

The rarity of GIST combined with nonspecific presentation frequently leads to delays in diagnosis [6]. Based on clinical presentation, several imaging techniques are available to support diagnosis: upper and lower endoscopy, GI contrast study, capsule endoscopy, double balloon enteroscopy, CT scan, CT enterography, and MRI [1, 7].

Successful treatment of GIST requires assessment of the extent and progression of disease [8], established by imaging studies. For patients with primary localized GIST, surgical resection with a negative microscopic margin (R0 resection) and an intact tumor pseudocapsule is the treatment of choice [8]. Since GIST rarely metastasize to lymph nodes, formal lymphadenectomy is not necessary. [8]. About 50% of patients with a complete resection of their primary localized GIST develop recurrent disease [8], so it is important to assess its risk of malignancy behaviour. The modified NIH method is a classification system of this risk, which is based on the tumor size, location of the tumor (stomach, small intestine, colon, rectum, or other), mitotic index (<5 or ≥5 mitoses per 50 HPF), and tumor rupture [1, 8, 9]. This classification system defines GIST as very low, low, intermediate, or high risk for recurrence [8]. Depending on risk stratification, specific molecular target therapy with imatinib may be necessary, which is also the first-line medical treatment for metastatic and unresectable GIST [8]. Our patient had a low risk GIST, so there was no need of further medical treatment.

In this case report, the authors emphasize a rare form of presentation of small intestine GIST with transient intussusception and intermittent GI bleeding. Clinicians should consider this diagnosis whenever dealing with obscure GI bleeding and/or intussusception, to provide early diagnosis and adequate treatment.
